# Mutational Frequencies in Mycobacterial *rpoB* gene using GeneXpert/MTB Rif Assay in Rifampicin Resistant patients at a tertiary care setting in Urban Sindh, Pakistan: Analysis from a Five-Year Period

**DOI:** 10.12669/pjms.37.4.3875

**Published:** 2021

**Authors:** Muhammad Alamgir, Mehwish Sajjad, Mirza Saifullah Baig, Muhammad Yahya Noori

**Affiliations:** 1 Muhammad Alamgir, M. Sc. Lab Technician, Ojha Institute of Chest Diseases, Dow University of Health Sciences, Karachi, Pakistan; 2 Mehwish Sajjad, MBBS, M. Phil. Lecturer, Department of Pathology, Dow International Medical College, Dow University of Health Sciences, Karachi, Pakistan; 3 Mirza Saifullah Baig, MBBS, FCPS. Assistant Professor of Pulmonology, Ojha Institute of Chest Diseases, Dow University of Health Sciences, Karachi, Pakistan; 4 Muhammad Yahya Noori, MBBS, Ph. D, MBA, M. Sc. Associate Professor of Microbiology, Department of Pathology, Dow International Medical College, Dow University of Health Sciences, Karachi, Pakistan

**Keywords:** *Mycobacterium Tuberculosis*, GeneXpert, Rifampicin resistance, *rpoB* gene, Probe Mutations

## Abstract

**Objectives:**

To assess the mutational frequencies in Mycobacterial *rpoB* gene using GeneXpert/MTB Rif Assay in rifampicin resistant patients during 2013-2017 at a tertiary care setting in Urban Sindh, Pakistan.

**Methods:**

This Retrospective Descriptive Cross-Sectional Study was conducted at the TB laboratories, Ojha Institute of Chest Diseases, Dow University of Health Sciences. The record of 713 positive cases of Rifampicin Resistant Tuberculosis from January 2013 to December 2017 were analysed. These were diagnosed using GeneXpert® that detects mutations in the 81 base pair region of *rpoB* gene with the help of five molecular probes A, B, C, D and E. All invalid and extra pulmonary samples were excluded.

**Results:**

In total, 713 cases were found to be rifampicin resistant during the five-year period, among which 374 (52.45%) were males while 339 (47.55%) were females. Among the five standard probes A, B, C, D and E, 97.48% of the cases had a single mutation. Among these, mutations in Probe E (66.48%) were the most common, followed by Probe B (14.3%) and Probe D (11.08%). Only 13 cases (1.82%) of double mutations and five cases (0.7%) of triple mutations were detected.

**Conclusion:**

The *rpoB gene* Probe E region 529-533 appears the most potent site for a mutation and development of rifampicin resistance in the *rpoB* gene in *Mycobacterium tuberculosis*, that encodes the β-subunit of RNA polymerase. The most affected age-group in both males and females is 19-45 Years.

## INTRODUCTION

Drug Resistant Tuberculosis (DR-TB) is a worldwide health problem. Not only it is difficult to treat but also the cost of treatment of DR-TB is much higher than the ordinary disease. It is unfortunate that Pakistan has the fourth highest prevalence of multi drug resistance and more than 15000 cases of DR-TB emerge every year.^1^

With the traditional Drug-Sensitivity Testing (DST) replaced by rapid molecular diagnostics, new areas of scientific inquiry are emerging in TB diagnostics and management. The GeneXpert MTB/RIF Assay is among the most popular tools, endorsed and recommended by the WHO,^2^ that detects rifampicin resistance in patient samples by detecting mutations in the *rpoB* gene. It uses a Real time PCR based closed system and detects changes in *rpoB* gene that encodes β subunit of RNA polymerase, the site where rifampicin binds and inhibits the polymerase from transcribing and stopping the protein production. It uses five different overlapping probes A, B, C, D and E to detect mutations in codons from 507-511, 511-518, 518-523, 523-529 and 529-533, respectively.^3^ These probes assist in differentiating among the wild type and mutated forms that are associated with rifampicin resistance. Different types of mutations have different effects on the bacterial physiology and survival, hence knowing the types, frequencies and geographical correlates of these mutations may influence future interventions.

As the first step, this study aims to determine the relative frequencies of different regional mutations through assessing the relative frequencies of associated probes for various *rpoB* gene mutations by GeneXpert MTB/RIF Assay.

## METHODS

It was retrospective cross-sectional study based on the diagnostic records of the Tuberculosis Laboratories of Dow University of Health Sciences at Ojha Institute of Chest Diseases. Ethical approval was obtained from the Institutional Review Board of Dow University of Health Sciences. All cases that were received and tested positive for rifampicin resistance on GeneXpert, during the five-year-period from 1^st^ January, 2013 to 31^st^ December, 2017 were included.

Data were analysed from 713 cases. Records were deidentified for names and only demographic details about age and gender were noted. All cases with invalid and extra pulmonary samples were excluded.

Samples were processed as per manufacturer’s instructions. Briefly, Xpert Sample Reagent (SR) was added to unprocessed sputum sample in a 2:1 ratio into a 15ml centrifuge tube and incubated at room temperature for 15 minutes. During incubation period, the samples were mixed by inverting the tubes gently 2 times every 5 minutes. Then 2.0 ml of liquefied sample was transferred to Xpert Cartridge (Ver 3.0) and loaded into GeneXpert IV machine and observed for results.^4^

Mutational frequencies were calculated for each probe. Data were tabulated in Microsoft Excel. Chi square test was applied using SPSS Version 24. Results were considered significant with p-value less than 0.05.

## RESULTS

Among the 713 cases analyzed for the study, 374 (52.45%) were males and 339 (47.55%) were females. The age distribution is summarized in Table-I.

**Table-I:** Gender-wise age distribution of rifampicin-resistant patients.

	0-18 Years	19-45 Years	>45 Years	Not Available*	Total
Males	36	243	79	16	374
Females	69	212	49	9	339
Total	105	455	128	25	713

* Patients, for whom age records were missing.

The most common group among the subjects was between 19 and 45 years. in both the genders. Gender-wise data were tabulated (Table-II) in relation to different probes from the 363 male and 332 female patients, who were resistant to rifampicin patients and the region examined by Probe E was found to be the most commonly affected by the mutation, followed by that of Probe B. Chi-square test showed differences to be statistically non-significant.

**Table-II:** Gender-wise distribution of rifampicin-resistance patients in relation to different regions of rpoB gene detected through Probes A, B, C, D and E.

Probe	Mutations		

	Male	Female	p-value
A	15	14	
B	56	46	
C	6	5	0.98
D	42	37	
E	244	230	
	363	332	

These data were further stratified for age group and probe type and it was observed that the age group 19-45 years had the highest number of mutations in the Probe E region. Data are summarized in Table-III.

**Table-III:** Age-wise distribution of rifampicin-resistance patients in relation to different regions of rpoB gene detected through Probes A, B, C, D and E

	0-18 Years	19-45 Years	>45 Years	Not Available	Totals
A	1	17	8	3	29
B	14	71	16	1	102
C	4	5	2	0	11
D	9	48	19	3	79
E	75	302	81	16	474

Totals	103	443	126	23	695

*Patients, for whom age records were missing.

A few patients (2.5%) with double and triple mutations were also found. Although the number was small, but interestingly some combinations were completely absent. Data are summarized in Table-IV.

**Table-IV:** Age -wise distribution of rifampicin-resistant patients having double or triple mutations in relation to different regions of rpoB gene detected through Probes A, B, C, D and E.

	0-18	19-45	>45	Not Available	Totals
AB	0	2	0	2	4
AD	1	6	0	0	7
DB	0	2	0	0	2
EAD	1	2	2	0	5

Totals	2	12	2	2	18

## DISCUSSION

Drug resistant Tuberculosis (DR-Tb) is among serious public health issues worldwide.^5^ It is considered a major threat to the developing countries. Pakistan has extremely high burden of tuberculosis patients, who are constantly facing the threat of developing drug resistance.^5^ Many diagnostic techniques are used for detection of TB and drug resistance, which now include rapid diagnostic tools such as GeneXpert. These can save precious time, which would otherwise be wasted through traditional methods to detect drug resistance.^6^ Due to limited requirements of infrastructure and technical expertise, GeneXpert is emerging as a particularly useful tool in the rural settings, which detects mutations in the 81bp region of 3534 bp *rpoB* gene with the help of five molecular beacons.^7^,^8^ The five probes used for this purpose have been shown to have different frequencies of mutations resulting in rifampicin resistance in studies from Pakistan, India, Bangladesh and other regions of the world, though mutations in probe E were found to be the highest in all studies, which is consistent with our results.^9^-^17^

Though the results from GeneXpert, do not provide the exact point of mutation in the *rpoB* gene, a number of sequencing studies show that codon 531, which is covered by Probe E is among the most common spots of mutation. Other specific mutations recognized in different studies were codon 526 (covered by Probe D) and 516 (covered by Probe B). The most conserved region, that has been found among different studies was the Probe C region, which is also consistent with our own findings.^9^-^17^ The mutation at codon 531, changes Serine to Leucine, changing the polar R group of Serine with the non-polar R group of Leucine, resulting in development of resistance to rifampicin.^18^ The highest frequency of this particular mutation may be explained by high mutagenic potential of Serine with downstream positive selection favouring this change, as it is considered as “a mutational hot-spot” and is the among the most frequently replaced amino acids.^19^ Further bioinformatics-based studies based upon this finding might provide interesting insights into this high frequency of mutation.

Some mutation combinations were also found where there were two or more mutations involving regions covered by probes, A, B: A, D: B, D and A, D, E, however, they were considerably less frequent, probably owing to the result of decreased selection pressure, once the organism has already acquired drug resistance through one of the mutations.

### Limitations of the study

Though our study had the limitations of not identifying the particular mutation directly through PCR and sequencing, however, the knowledge about probe frequency can be used to screen for the trends of evolving rifampicin resistance in *Mycobacterium tuberculosis*. In addition our other limitations include side-by-side comparison with culture and drug susceptibility testing, to rule out false negative and positive results for GeneXpert.

## CONCLUSION

This study showed that the mutations in the Probe E region are disproportionately common, while the age-group 19-45 was the most commonly affected age-group among the rifampicin-resistant patients.

### Author Contribution:

**MA:** Performed the GenXpert/MTB Rif Assay and recorded the data.

**MS:** Wrote the first draft, compiled and tabulated data.

**SB:** Conceived the idea, provided clinical supervision, Edited the manuscript.

**MYN:** Conceived the idea, designed the project, provided laboratory and overall supervision, prepared the final draft of the manuscript. He is also responsible for the study.
